# Low length-for-age Z-score within 1 month after birth predicts hyperdynamic circulation at the age of 21 years in rural Malawi

**DOI:** 10.1038/s41598-023-37269-9

**Published:** 2023-06-24

**Authors:** Roosa-Maria Penninkangas, Manoj Kumar Choudhary, Charles Mangani, Kenneth Maleta, Tiina Teivaanmäki, Onni Niemelä, Per Ashorn, Ulla Ashorn, Ilkka Pörsti

**Affiliations:** 1 Faculty of Medicine and Health Technology, Tampere University, 33014 Tampere, Finland; 2 School of Global and Public Health, Kamuzu University of Health Sciences, Chichiri Blantyre, Malawi; 3 Department of Pediatrics, Helsinki University Hospital, Helsinki, Finland; 4 Department of Laboratory Medicine and Medical Research Unit, Seinäjoki Central Hospital, Seinäjoki, Finland; 5 Department of Pediatrics, Tampere University Hospital, Tampere, Finland; 6 Department of Internal Medicine, Tampere University Hospital, Tampere, Finland

**Keywords:** Cardiology, Cardiovascular diseases

## Abstract

Low birth weight predisposes to the development of hypertension in middle- and high-income countries. We examined the relation of early life length-for-age score (Z-score) on cardiovascular function in young adults in Malawi, a low-income country. Capture of supine, seated, and standing brachial pulse waveforms (Mobil-O-Graph) were performed in 223 females and 152 males (mean age 21 years), and analyzed according to the length-for-age Z-score tertiles during the first month of life. Plasma LDL cholesterol in young adulthood was slightly lower in the lowest versus highest tertile. Otherwise, blood hemoglobin and plasma chemistry were similar in all tertiles. Irrespective of posture, blood pressure, forward and backward wave amplitudes, and pulse wave velocity were corresponding in all tertiles. In the three postures, the lowest tertile presented with 4.5% lower systemic vascular resistance than the highest tertile (p = 0.005), and 4.4% and 5.5% higher cardiac output than the middle and highest tertiles, respectively (p < 0.01). Left cardiac work was 6.8% and 6.9% higher in the lowest tertile than in the middle and highest tertiles, respectively (p < 0.01). To conclude, in a low-income environment, low length-for-age Z-score after birth predicted hyperdynamic circulation at 21 years of age without changes in blood pressure and metabolic variables.

## Introduction

People living in low-income countries have on average better overall cardiovascular risk profile than people in high-income countries, but the incidence and mortality from cardiovascular diseases are higher in low-income countries^1^. This may be attributed to nutritional factors, higher burden of infections, lower level of education, and suboptimal health care services^1,2^. Underweight and growth stunting are common problems among children in low-income African countries^2^. According to the United Nations Children’s Fund (UNICEF) data from Malawi in 2019–2020, 13% of the newborn babies had low birth weight^3^.

Fetal undernutrition can have a permanent influence on tissue structure and physiology^4^. Early life undernutrition may induce changes in gut microbiota that predispose to growth faltering, as the transfer of microbiota from undernourished Malawian children into germ-free mice resulted in transmission of the impaired growth phenotype to the experimental animals^5^. Infancy is a critical developmental period, during which severe malnutrition may influence metabolic capacity in the long term and confer elevated susceptibility to non-communicable diseases^6^. Growth restriction can affect children’s cognitive and motor development^7^, but has also potential influences on the cardiovascular system^4,8^. In Malawi, 36% of all children under 5 years of age present with growth stunting^3^, while the prevalence of wasting (low weight-for-height) is 3%^3^, which is still within the acceptable range (< 5%) according to the WHO criteria^2^.

According to the Barker hypothesis, early life undernutrition predisposes to disturbances of glucose and lipid metabolism, obesity, coronary heart disease, and high blood pressure (BP) that emerge in adulthood^8^. Such pathophysiological changes can take place despite adequate catch-up growth during later childhood when energy-rich nutrition is available^8^. In 272 Malawian hospitalized children aged 0.5–5 years, malnutrition was associated with lower BP and systemic vascular resistance, whilst increased cardiac output was observed in the most severely wasted children^9^.

Currently, oscillometric recordings of BP and pulse waveforms from the brachial artery allow for the derivation of various hemodynamic variables^10–16^. These variables encompass aortic BP^10,17^, forward and backward wave amplitudes^10,12^, cardiac output, and total peripheral resistance^14,18,19^. Moreover, devices like the Mobil-O-Graph can estimate pulse wave velocity (PWV) by comparing the time delay between the pulse waveforms evaluated at the aortic and brachial sites^15,20^. Assessment of cardiac output^18,19^ and wave reflections^11,12^ using this method have provided values of acceptable accuracy. Overall, this approach provides a reasonable compromise between accuracy and ease of use.

Limited information exists from low-income countries regarding the impact of early-life growth on cardiovascular function in adulthood. From 1995 to 1996, a total of 795 pregnant women attending the Lungwena health center antenatal clinic in Malawi were enrolled in a longitudinal study to track their descendants^21^. Previous reports have been published on the follow-up^22–26^, and it is still ongoing. In this cohort, the prevalence of underweight, growth stunting, and wasting reached its highest point between 3 and 21 months of age within the first three years of life^23,27^. We conducted a study on 375 individuals from this cohort, recording their brachial BPs and oscillometric pulse waveforms, to investigate whether early-life body size influences their hemodynamic variables at the age of 21 years.

## Methods

The present cohort has been followed prospectively since their birth in Lungwena, a rural area in Southern Malawi. The original participants were 795 pregnant mothers visiting the Lungwena health center between June 1995 and August 1996. The distance to the nearest town, Mangochi, was 30–40 km, and no commercially manufactured food products were available at local shops. The livelihoods of the participants were dependent on rain-fed small-scale agriculture, fishing, and petty trading. Based on 24-h dietary recall, maize provided two-thirds (63%) of the dietary energy in Lungwena, while other energy sources were roots and tubers (11%), fish (5%), fruit (4%), legumes (4%) and vegetables (3%)^22^. The number of children born alive was 767^26^, and they were recruited to the follow-up. By the age of 15 years, 179 children from the cohort had deceased^25^.

When the subjects were ~ 21 years of age, they were invited to participate in brachial pulse waveform analyses. Successful recordings were performed on 419 from in total 429 participants^27^. Information about weight and length at birth and at one month was available from 375 participants: 223 females (age range 20.4–22.3 years) and 152 males (age range 20.4–22.2 years). The average values of body size during these two measurements were used in the analyses. The recruitment, data collection, and follow-up have been described previously^26^. Length and height values were converted to Z-scores using the WHO Multi-Center Growth Reference Study for children data^28^.

Ethical approval for the original Lungwena Child Survival Study (LCSS) was obtained from the National Health Science Research Committee in Malawi (HSRC 93⁄94), while approval for the present follow-up study was obtained from the College of Medicine Research and Ethics Committee (COMREC) on the 31st of October 2016. The study complies with the Declaration of Helsinki. Informed consent was acquired verbally from all caregivers of the children in the beginning of the study^21^. Later, informed consent was obtained again from the guardians and the children before the study visits until the age of 15 years^25,26^, and from the present participants when they were ~ 21 years of age. The consents were acquired by a person living in Lungwena and speaking the local dialect, and the participants either signed the consent or confirmed the document with their fingerprint image.

### Laboratory analyses

Blood hemoglobin concentration was determined using HemoCue (Angelholm, Sweden). Malaria was screened using SD Bioline Malaria Ag Pf test (Standard Diagnostic Inc., South Korea) specific to *Plasmodium falciparum* (sensitivity 99.7%, specificity 99.5%). Plasma was separated and stored at − 70 °C until cold-shipped to Finland. The laboratory analyses were performed at the Medical Research Unit, Seinäjoki Central Hospital, Seinäjoki, Finland (SFS-EN ISO/IEC 15189:2013 accredited unit). Plasma C-reactive protein, glucose; total, high-density lipoprotein (HDL), low-density lipoprotein (LDL) cholesterol; triglycerides, sodium, potassium, and creatinine were determined using Cobas c702 or Cobas c702 ISE photometric assay tests (F. Hoffmann-Laroche Ltd, Basel; Switzerland). Glomerular filtration rate (eGFR) was estimated using a creatinine-based formula^29^.

### Blood pressure and analyses of brachial pulse waveforms

An automated oscillometric device (Mobil-O-Graph, I.E.M., Stolberg, Germany) was applied to measure brachial BP and perform waveform-derived evaluation of aortic BP, wave reflections, PWV, heart rate, stroke volume, cardiac output, and systemic vascular resistance^27^. The last three values were normalized to body surface area and expressed as stroke index, cardiac index, and systemic vascular resistance index (SVRI). The formula 0.01439 × (mean aortic pressure − pulmonary artery occlusion pressure) × cardiac index was applied to calculate left cardiac work index (LCWI)^30–32^. Normal pulmonary artery occlusion pressure was assumed (6 mmHg), while 0.0143 is the conversion factor of pressure from mmHg to centimeters of water, volume to density of blood (in kg/L), and centimeters to meters. The brachial cuffs were chosen according to the upper arm circumference. The Mobil-O-Graph has been validated for measurement of peripheral and central BP^16,33^ and 24-h ambulatory BP^17,34^. Moreover, the evaluated PWV has been compared with tonometrically and invasively acquired values with acceptable accuracy^35^. The recordings were performed by the Lungwena research team that had ~ 20 years of experience in child health-related research.

### Experimental protocol

As posture can individually influence BP, and especially diastolic BP increases when changing from the supine to the seated or standing positions^27,36–38^, the present Mobil-O-Graph recordings were performed in supine, seated, and standing postures in a quiet room. (1) The brachial BP cuff was attached, and after ~ 5 min in the seated position, two measurements were taken. (2) The subjects rested supine for ~ 5 min before the third recording was performed. (3) The subjects stood up and the fourth and the fifth measurements were performed after ~ 5 min of standing. We have previously found that the hemodynamic variables remain quite stable after about two minutes in either supine or upright posture^31,32,36^. The supine-to-upright change in body position complied with the protocol to examine orthostatic BP responses^39^. The figures present the results in the order supine-seated-standing.

### Statistics

The participants were divided to sex-specific tertiles according to the length-for-age Z-score during the first month of life. The characteristics between the tertiles were analyzed using analysis of variance (ANOVA) followed by t-test for normally distributed variables, and Kruskal–Wallis test followed by Mann–Whitney U-test for variables with skewed distribution. Tertile proportions were compared using Chi-square test. The homogeneity of variances was tested with the Levene’s test, the Bonferroni correction was applied for all post-hoc analyses.

Hemodynamic differences between the tertiles in the supine, seated and standing postures were examined using generalized estimating equations (GEE) to examine the influences of tertile membership, posture, and their interaction on the variable of interest. The linear scale response was selected, and the autoregressive option was chosen, as the serial measures of hemodynamic variables are autocorrelated. As the tertiles presented with differences in gestation length and plasma concentration of LDL cholesterol (Table 1), the analyses were adjusted for these differences by including the variables as covariates. The PWV analyses were additionally adjusted for mean aortic pressure^40,41^.

**Table 1:** Demographic and anthropometric data according to the tertiles of the length-for-age Z-score during the first month of life.

	Lowest tertile (n = 125)	Middle tertile (n = 124)	Highest tertile (n = 126)
Length for age Z-score during the 1st month	− 2.79 (0.75)	− 1.43 (0.27)	− 0.27 (0.55)
Length during the 1st month (cm)	47.3 (2.1)	49.7 (1.3)	51.8 (1.8)
Weight-for-age Z-score during the 1st month	− 1.46 (1.02)	− 0.48 (0.79)	− 0.08 (0.78)
Number, male/female	53/72	47/77	52/74
Variables at the time of haemodynamic studies			
Age (years)	21.3 (0.4)	21.3 (0.4)	21.3 (0.5)
Height (cm)	158 (7)	160 (7)	163 (8)
Weight (kg)	51.9 (6.9)	54.5 (6.9)	57.1 (7.6)
Mean seated systolic BP (mmHg)	121 (12)	119 (11)	121 (11)
Mean seated diastolic BP (mmHg)	76 (9)	74 (7)	75 (8)
BMI (kg/m^2^)	20.9 (2.3)	21.2 (2.1)	21.6 (2.1)

Data are mean (standard deviation).

*BP* blood pressure, *BMI* body mass index.

The results were presented as mean and standard deviation (SD) or as median [25th–75th percentile] in the text and tables, and as mean and standard error of the mean (SEM) in the figures, and *p* < 0.05 was considered statistically significant. SPSS version 26.0 (IBM SPSS Statistics, Armonk, NY, USA) was used.

## Results

### Study group

The demographic and anthropometric data according to the tertiles of the length-for-age Z-score are presented in Table 1. During the first month of life body length (p < 0.001) and weight-for-age Z-score (p < 0.001) were lowest in the lowest tertile, and lower in the middle tertile than in the highest tertile (p < 0.001 for both). The proportion of females ranged from 57.6 to 62.1% without significant difference between the tertiles (p = 0.989). At 21 years of age, mean seated brachial BP was similar in all tertiles: 121, 119, and 121 mmHg for systolic BP (p = 0.456), and 76, 74, and 75 mmHg for diastolic BP (p = 0.298), in the lowest, middle, and highest tertiles, respectively. However, small differences in height and weight persisted (p < 0.05 for all between-tertile comparisons), while body mass indexes were not statistically significantly different (p = 0.054) in the tertiles.

### Laboratory data

Hemoglobin, prevalence of *Plasmodium falciparum* positivity in the rapid test, and estimated glomerular filtration did not differ between the tertiles (p > 0.435 for all) (Table 2). Plasma creatinine, sodium, potassium, urid acid, glucose, C-reactive protein, alanine aminotransferase, alkaline phosphatase, total and HDL cholesterol, and triglyceride concentrations did not differ between the tertiles, either (p > 0.053 for all). LDL cholesterol was 0.18 mmol/l higher in the highest versus the lowest tertile (p = 0.034) (Table 2).

**Table 2:** Laboratory data according to the tertiles of the length-for-age Z-score during the first month of life.

	Lowest tertile (n = 125)	Middle tertile (n = 121)^†^	Highest tertile (n = 125)^‡^	p-value
Hemoglobin (g/l)	143 (17)	141 (19)	144 (19)	0.508
Positive rapid test for *Plasmodium falciparum* (n)	18	25	23	0.444
eGFR (ml/min/1.72 m^2^)	131 (9)	129 (13)	129 (12)	0.436
Creatinine (µmol/l)	58 (13)	59 (13)	60 (13)	0.633
Sodium (mmol/l)	138 (2)	138 (2)	138 (3)	0.740
Potassium (mmol/l)	4.0 (0.4)	4.0 (0.4)	4.0 (0.4)	0.908
Uric acid (µmol/l)	281 (70)	271 (73)	273 (76)	0.502
Glucose (mmol/l)	5.0 (0.8)	4.8 (0.6)	5.0 (0.6)	0.054
CRP (mg/l)	0.8 [0.4–1.6]	1.0 [0.5–2.0]	0.9 [0.4–2.3]	0.381
ALT (U/I)	26 (13)	30 (28)	25 (12)	0.070
ALP (U/I)	103 (38)	107 (49)	103 (43)	0.674
Total cholesterol (mmol/l)	3.1 (0.7)	3.2 (0.6)	3.3 (0.7)	0.067
HDL cholesterol (mmol/l)	1.0 (0.3)	1.0 (0.3)	1.0 (0.3)	0.942
LDL cholesterol (mmol/l)	1.65 (0.6)	1.74 (0.5)	1.83 (0.6)*	0.041
Triglycerides (mmol/l)	0.8 [0.6–1.0]	0.8 [0.6–1.1]	0.8 [0.6–1.0]	0.904

Data are mean (standard deviation) or median [25th–75th percentile].

*eGFR* estimated glomerular filtration rate using the CKD-EPI creatinine-based formula, *CRP* C-reactive protein, *ALT* alanine aminotransferase, *ALP* alkaline phosphatase, *HDL* high-density lipoprotein, *LDL* low-density lipoprotein.

^†^Total participant number was 124, but laboratory results were missing from 3 participants.

^‡^Total participant number was 126, but laboratory results were missing from 1 participant.

*p = 0.034 versus lowest tertile.

### Hemodynamic results

**Figure 1:**
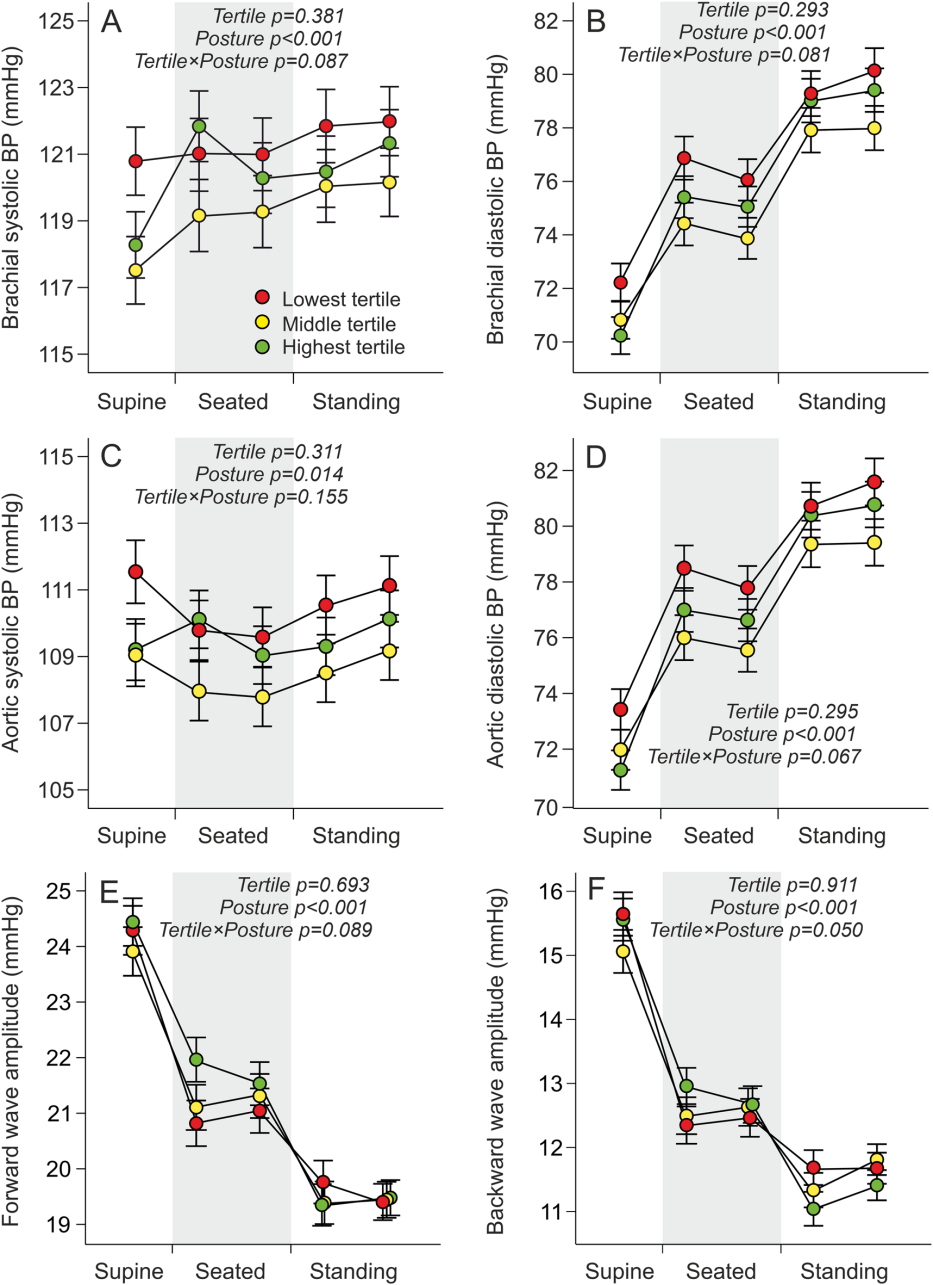
Line graphs show brachial systolic (**A**) and diastolic (**B**), and aortic systolic (**C**) and diastolic (**D**) blood pressure, and forward wave amplitude (**E**) and backward wave amplitude (**F**) according to the tertiles of the length-for-age Z-score during first month of life during supine, seated and standing positions; lowest tertile n = 125 when supine and seated, n = 123 when standing; middle tertile n = 124 when supine and seated, n = 122 when standing; highest tertile n = 126 when supine and seated, n = 125 when standing; mean ± standard error of the mean. Statistics by generalized estimating equations with adjustments for plasma concentration of LDL cholesterol; *p* values denote differences between tertiles.

**Figure 2:**
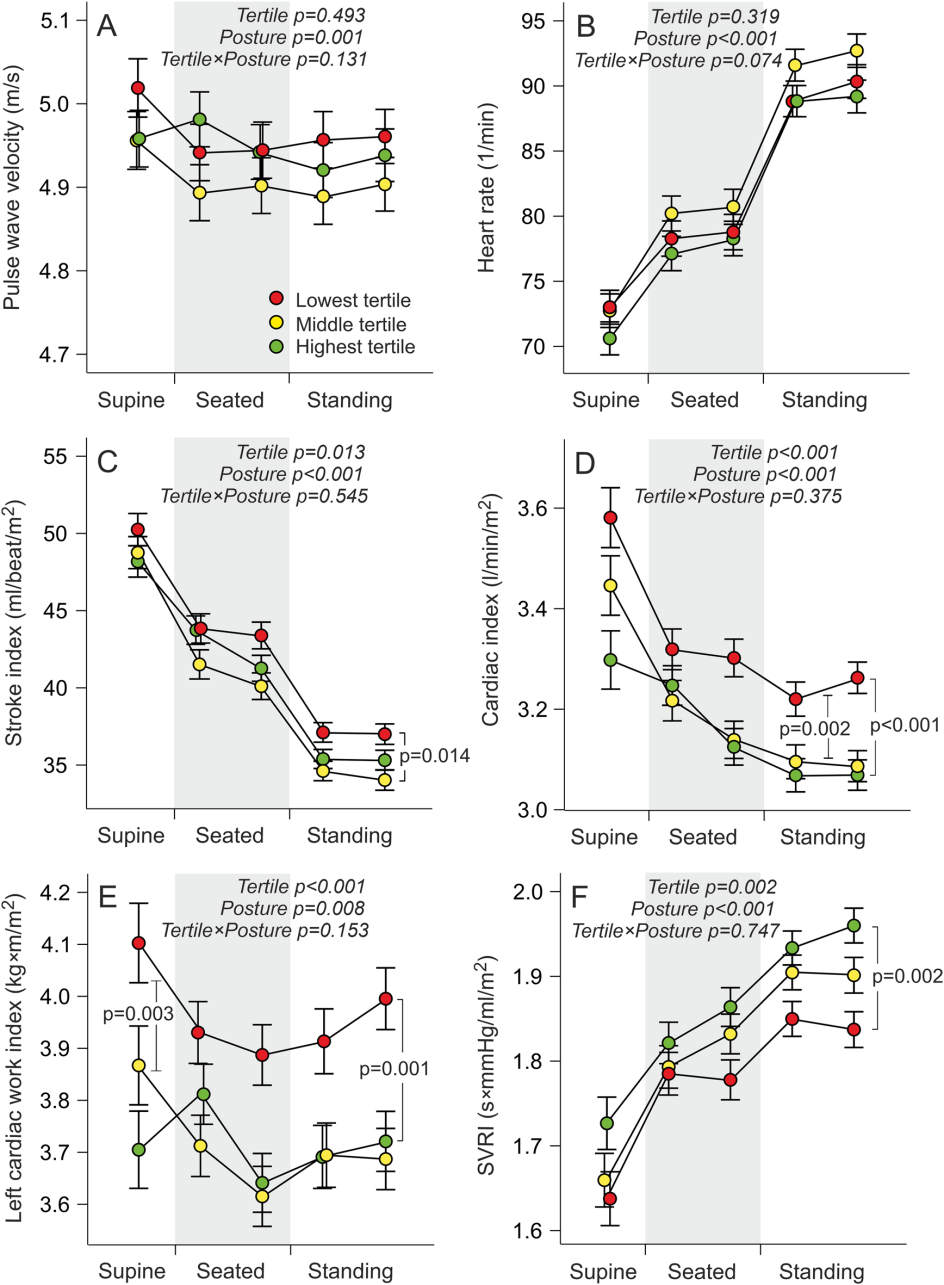
Line graphs show pulse wave velocity (**A**), heart rate (**B**), stroke index (**C**), cardiac index (**D**), left cardiac work index (**E**), and systemic vascular resistance index (SVRI) (**F**), division to tertiles and statistics as in Figure, pulse wave velocity was additionally adjusted for mean aortic blood pressure.

Oscillometric brachial pulse waveforms were recorded in supine, seated, and standing postures (Figs. 1, 2). No differences between the tertiles were observed in brachial and aortic systolic and diastolic BP (Fig. 1A–D), forward wave amplitude (Fig. 1E), backward wave amplitude (Fig. 1F), and augmentation index (*p* = 0.442, data not shown). All these variables changed significantly in response to change in posture, while no significant interactions between the tertiles and posture were found (Fig. 1A–F).

PWV (Fig. 2A) and heart rate (Fig. 2B) did not differ between the tertiles, while both variables changed in response to posture without significant interactions between the tertiles. Stroke index progressively decreased from supine to seated, and from seated to standing postures, while no significant tertile-posture interactions were observed. However, the lowest tertile presented with higher stroke index than the middle tertile, the average difference though postures being 2.7 ml/min/m^2^ (p = 0.014) (Fig. 2C). Cardiac index and LCWI also decreased from supine to seated, and from seated to standing positions in all tertiles without significant tertile-posture interactions (Fig. 2D,E). On average, cardiac index was 0.15 l/min/m^2^ higher in the lowest than in the middle tertile (p = 0.002), and 0.20 l/min/m^2^ higher in the lowest than in the highest tertile (p < 0.001). Mean LCWI was 0.26 kg × m/m^2^ higher in the lowest than in the middle tertile (p = 0.003), and 0.29 kg × m/m^2^ higher in the lowest than in the highest tertile (p = 0.001). SVRI gradually increased from the supine to the seated and standing postures in all tertiles without significant tertile-posture interactions. Average SVRI was 0.10 s × mmHg/ml/m^2^ lower (p = 0.002) in the lowest tertile than in the highest tertile (Fig. 2F).

To eliminate the influence of differences in body size on the cardiac variables and vascular resistance, the above results were adjusted for body surface area. The following comparison emphasizes the significance of this adjustment: The present young women were smaller than men, and the respective body surface areas were 1.50 (0.12) m^2^ vs. 1.63 (0.11) m^2^ (p < 0.001). Estimated cardiac output was lower in women than in men 4.69 (0.45) vs. 5.12 (0.55) l/min (p < 0.001). However, when related to body surface area the cardiac index values were identical between women and men: 3.14 (0.35) vs. 3.14 (0.34) l/min/m^2^, respectively.

## Discussion

To examine the influence of early life size on cardiovascular function in young adults from a low-income country, the present participants from rural Malawi were divided to tertiles according to the length-for-age Z-score during their first month of life. Small early-life body size was reflected in the hemodynamics of these subjects at 21 years of age, as in the lowest tertile cardiac index and cardiac workload irrespective of posture, as evaluated by LCWI, were higher than in the other two tertiles. There were no differences in brachial or central BP between the tertiles, probably due to the lower SVRI in the lowest tertile that reached statistical significance when compared with the highest tertile.

Previously, low birth weight (n = 20) was associated with higher risk of elevated BP among 77 Swedish men who were examined at the age of 28 years (odds ratio 3.63), and the authors concluded that being born small for gestational age may be a predictor for raised BP in adult life^42^. In 4–18-year-old children and adolescents in Spain (n = 630), the participants with lowest birth weights (n = 35) had highest 24-h ambulatory BP values in the absence of differences in heart rate^43^. However, contradictory findings have also been reported, showing no significant differences in laboratory BP in children and teenagers aged 7–18 years (n = 219), despite differences in birthweight^44^. In the present study we did not observe differences in brachial or aortic BP between the tertiles of the length-for-age Z-score in the supine, seated or standing postures. BP assessment in three postures was included in the measurements, as exaggerated systolic BP response to standing was recently found to be an independent predictor of adverse cardiovascular events^45^. Moreover, we included aortic BP in the analyses, as central BP is considered to be a more reliable predictor of cardiovascular risk than brachial BP^46,47^.

In the absence of differences in BP, the lowest tertile in this study presented with lower SVRI than the highest tertile, and higher cardiac index than the two other tertiles, while the laboratory analyses did not show evidence of unfavorable harmful changes in the metabolic profiles. Thus, young Malawian adults with small stature at birth had hyperdynamic circulation at the age of 21. This was also translated to higher LCWI, which cannot be regarded as a favorable hemodynamic finding. The long-term influences of increased cardiac workload during normotension remain rather unknown. Hyperdynamic circulation may be an early feature of essential hypertension, which predisposes to the development of established hypertension and progression of vascular disease^48^. In the Bogalusa Heart Study, hyperdynamic circulation, as assessed using pulse pressure and heart rate measurements, was associated with adverse cardiovascular risk profile among children aged 8 to 17 years^49^. Low systemic vascular resistance and high cardiac output are also detected in patients with high-output cardiac failure, who have high metabolic rate, while the chronic increases in cardiac output in these patients eventually lead to a progressive decline in cardiac function^50^. Of note, higher left cardiac work in the upright posture, but not in the supine position, has been previously reported in both Malawian and Finnish men when compared with women^27,32^.

Backward wave amplitude and augmentation index reflect the influence of wave reflection on central pulse pressure^51^. Wave reflection is influenced by arterial stiffness, heart rate, stroke volume, and systemic vascular resistance^51,52^. Arterial stiffness increases with ageing, leading to an earlier return of the reflected waves which increases the central augmentation of pulse pressure^51^. The PWV values evaluated by the Mobil-O-Graph did not differ between the study tertiles and the values were also within the normal limits of a large European reference population^53^. The amplitude of the reflected wave is associated with hypertensive end organ damage and it may also predict future cardiovascular events^54^. Among 7–18 year-old Spanish children and teenagers (n = 219) highest augmentation index values were recorded from subjects born with low birth weight^44^. However, in the present study backward wave amplitude and augmentation index at 21 years did not differ between the tertiles of the length-for-age Z-score during the first month of life.

Malnutrition, inflammation, infections, low-income, and low socio-economic status are factors that may influence growth and cardiovascular function^1,55,56^. Babies in Malawi have been reported to be born about 2.5 cm shorter with about 500 g lower body weight than babies in a high-income reference population^23,24^. Among the pregnant mothers of the present study subjects the seroprevalence of human immunodeficiency virus (HIV) infection was high (18%)^57^. Moreover, impaired linear growth, high incidence of growth stunting (~ 70%) and underweight were observed among the study cohort that peaked at 3–21 months of age^23,24^. Morbidity in infancy, small size after birth, maternal HIV infection, and home delivery were the strongest predictors of severe underweight in these subjects^23^. By the age of 15 years, 179 subjects of the present cohort had already been deceased^25^. The Mangochi area is characterized by high malaria transmission due to high temperatures and frequent rainfalls from October to April^58^. Reflecting this endemic situation, 17–18% of the present young men and women were tested positive for Falciparum malaria^58^. However, the mean results of the laboratory tests reflecting inflammation, electrolyte balance, glucose metabolism, renal function, and lipid profiles were well within the normal range in all study tertiles. Also, low length-for-age and weight-for-age Z-scores during the first month of life were not related with adverse metabolic changes at young adulthood. Altogether, the present results suggest that in the absence of energy-rich nutrition, the Barker hypothesis may not apply to young adults in a low-income country.

We evaluated cardiovascular function by the use of an oscillometric device that has been validated for the measurement of peripheral and central BP^16,34^. Previously, successful comparisons between the evaluated PWV values and tonometrically and invasively obtained values have been reported^16,34,35^. However, criticism concerning the reliability of the PWV measurement by Mobil-O-Graph has been presented^59^, and therefore the results must be interpreted with caution. A further limitation is that the cardiac output measurements in 24 hospitalized patients using Mobil-O-Graph presented with slightly lower values than the thermodilution method^18^. However, the values were reproducible and the accuracy of the device was considered to be acceptable^18^. The cardiac output measurement also corresponded well to values measured using transthoracic echocardiography^14^. In the present analyses comparing the study tertiles, cardiac output, left cardiac work, and systemic vascular resistance were adjusted for body surface area. This approach is a strength of our study, as it eliminates the deviations caused by differences in body size among the participants.

In conclusion, young Malawian adults with low length-for-age and weight-for-age Z-scores after birth presented with hyperdynamic circulation and increased left ventricular workload in the absence of changes in BP or metabolic profiles at the age of 21 years.

## Data Availability

Access to the deidentified data of this study can be provided upon reasonable request to the corresponding author from qualified researchers who are adequately experienced in human subject confidentiality protocols.
